# Decreases in Arterial Stiffness and Wave Reflection after Isometric Handgrip Training Are Associated with Improvements in Cognitive Function in Older Adults

**DOI:** 10.3390/ijerph19159585

**Published:** 2022-08-04

**Authors:** Takanobu Okamoto, Yuto Hashimoto

**Affiliations:** 1 Department of Exercise Physiology, Nippon Sport Science University, Tokyo 158-8508, Japan; 2 Research Institute for Sport Science, Nippon Sport Science University, Tokyo 158-8508, Japan

**Keywords:** pulse wave velocity, augmentation index, blood pressure, trail-making test, carotid blood flow

## Abstract

This study aimed to investigate whether decreases in arterial stiffness and wave reflection after isometric handgrip (IHG) training improve cognitive function in older adults. Twenty-two older adults (mean age ± standard error: 75 ± 2 years) were randomly assigned to either a group that performed IHG training (IHG group, *n* = 11) or a sedentary control group (CON group, *n* = 11). The IHG exercise comprised four unilateral 2-minute isometric contractions at 30% of maximal voluntary contraction using a programmed handgrip dynamometer with 1-minute rest periods, performed 5 days per week for 8 weeks. Carotid pulse wave velocity (cPWV) and carotid augmentation index (cAIx) were measured, and the trail-making test (TMT) parts A (TMT-A) and B (TMT-B) were performed before (baseline) and after 8 weeks of training in both groups. After 8 weeks of training, cPWV, cAIx, TMT-A, and TMT-B were significantly reduced in the IHG group (*p* < 0.05). Significant positive correlations were found between the amount of change in cPWV and cAIx and that in TMT-A (*p* < 0.05 each). In addition, positive correlation trends were observed between the amount of change in cPWV and cAIx and that in TMT-B (*p* = 0.06, *p* = 0.05, respectively). The results of the present study suggest that IHG training-induced decreases in arterial stiffness and wave reflection are associated with improvements in cognitive function in older adults.

## 1. Introduction

The average age of the population is increasing worldwide and, accordingly, the prevalence of dementia [1]. A decline in cognitive function is an important health concern associated with a decreased quality of life. Stiffness of large elastic arteries, such as the carotids and the aorta, is a strong and independent risk factor for early cognitive impairment and dementia [2]. In fact, previous studies have reported an inverse relationship between pulse wave velocity (PWV) and cognitive function in older adults [3,4], suggesting that stiffer arteries may reduce cognitive function. The brain requires constant blood flow, and reductions in cerebral blood flow lead to cognitive problems. Cerebral blood flow is maintained relatively constant owing to cerebrovascular autoregulation even when arterial pressure changes, at least within a certain range [5]. Stiffening of the central arteries increases pulsatile stress, which is manifested in cerebrovascular microcirculation [6]. This stress can increase the tendency for microvascular injury and remodeling in the brain, leading to impaired cognitive function [7]. A recent meta-analysis reported that higher arterial stiffness is associated with biomarkers for cerebral small-vessel disease and cognitive problems [6]. Therefore, prevention of microvascular injury in the brain and maintenance of cognitive function may require reductions in arterial stiffness.

Isometric handgrip (IHG) training may be an important non-pharmacological therapy to reduce central and peripheral arterial stiffness and blood pressure (BP) [8,9,10,11,12]. Recently, Okamoto et al. [13] showed that IHG training reduces central and peripheral arterial stiffness and BP in older adults. Therefore, IHG training may be effective for preventing cognitive decline and impairment due to reductions in microvascular damage caused by decreases in arterial stiffness and BP.

The purpose of present study was to investigate the effects of decreased central arterial stiffness after IHG training on cognitive function in older adults. We hypothesized that reductions in central arterial stiffness after IHG training would be associated with improvements in cognitive function in older adults.

## 2. Materials and Methods

### 2.1. Study Design

This was a quasi-experimental control group study.

### 2.2. Subjects

**Figure 1:**
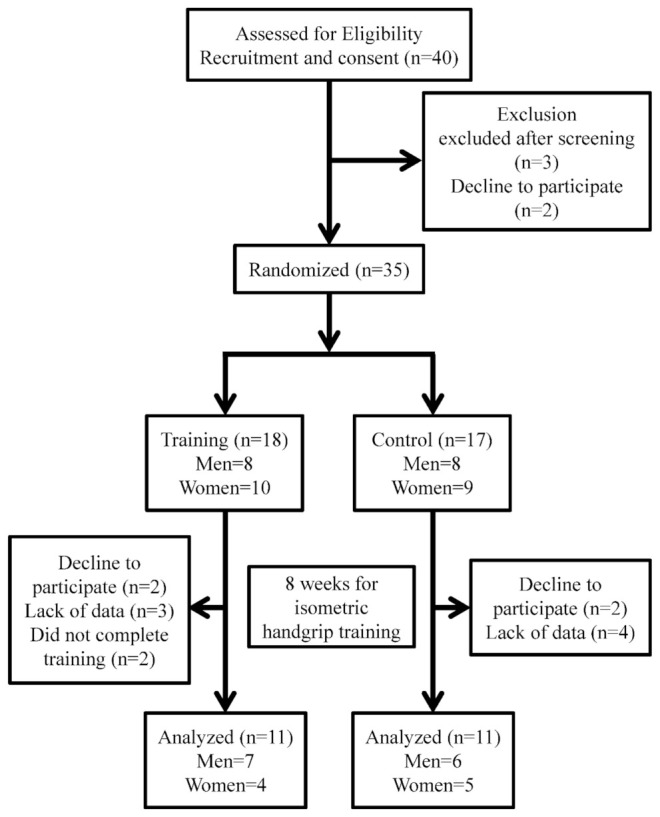
CONSORT flow diagram of the study participants.

A flow diagram summarizing the study enrollment and group assignments is shown in Figure 1. In total, 35 community-dwelling older adults (mean age ± standard error: 73 ± 2 years) who had not engaged in regular exercise (defined as ≤1 h/week) over the previous 2 years were enrolled. The participants’ physical activity levels were evaluated using the International Physical Activity Questionnaire—Short Form. None of the enrolled participants reported having a history of cardiovascular disease. The inclusion criteria were as follows: nonsmoker; non-obese (defined as a body mass index (BMI) ≤25 kg/m^2^); age ≥ 65 years; no diseases or disorders that could affect physical activity levels; not currently taking any medications that could compromise the cardiovascular system (e.g., antihyperlipidemic, antihypertensive, or antihyperglycemic agents); and not currently undergoing hormone replacement therapy. The exclusion criteria were as follows: obese (defined as a BMI >25 kg/m^2^); current smoker; presence of cardiovascular disease or diabetes; presence of dyslipidemia (defined as triglycerides (TG) ≥150 mg/dL, high-density lipoprotein cholesterol (HDL-C) <40 mg/dL, and low-density lipoprotein cholesterol (LDL-C) ≥140 mg/dL) [14]; or still menstruating. The medical history of each participant was confirmed by the authors during screening before the experiment. Table 1 shows the participants’ physical characteristics and serum lipid profiles at baseline. After screening was complete, the participants were randomly assigned to either a group that performed IHG training (IHG group, *n* = 11) or a sedentary control group (CON group, *n* = 11). The ethics committee of Nippon Sport Science University approved the protocol of the present study, which was performed in accordance with the Declaration of Helsinki and the guidelines published by our Institutional Review Board for experimental studies involving humans (017-H061). Detailed explanations of the potential risks associated with participation in the study were given to all participants, after which written informed consent was obtained.

### 2.3. Body Composition

Before the other measurements, body composition was determined by bioelectrical impedance analysis (InBody, Biospace, Seoul, Korea).

### 2.4. Measurements

All measurements were obtained twice: once at baseline and again at the end of the 8-week study period. After having the participants rest in the supine position for at least 20 min in a quiet room at a constant temperature (23–25 °C), carotid-heart PWV (hcPWV), carotid augmentation index (cAIx), carotid systolic BP (cSBP), and brachial BP were measured in addition to blood sampling and administration of the trail-making test (TMT). To help avoid potential diurnal variation, throughout the entire study period, the participants were tested at the same time of day. In addition, all participants were asked to abstain from intense physical activity within 48 h of the beginning of the study. Moreover, to eliminate the potential effects of food intake, BP, cAIx, and other measurements were taken after overnight fasting. All parameters were measured by the same investigator, who was blinded to the group assignment of the participants.

### 2.5. hcPWV, cAIx, and cSBP

hcPWV is a measure of carotid arterial stiffness and was assessed as previously reported [15]. Arterial applanation tonometry with PWV/ABI micro piezo-resistive transducers (Omron-Colin, Kyoto, Japan) was used to measure carotid artery pressure waveforms for 30 s. These transducers contain 15 pressure-sensitive components that are oriented side-by-side to detect carotid pulse traces. The transducers were affixed adjacent to the left common carotid arteries. The tonometry sensor for the carotid artery was attached to the neck with a clip. We measured the carotid pressure wave by calibrating the brachial pressure wave, while considering that the mean diastolic BP (DBP) was identical at both sites. We calculated the mean BP (MBP) on the carotid pressure wave and considered it to be the same as the brachial MBP (bMBP) in the corresponding heartbeat. We calculated the carotid pressure amplitude from the DBP and the location of the MBP on the carotid pressure wave [16]. We obtained the average of the brachial systolic BP (bSBP) and cSBP for waves recorded for 10 s. The interval between the heart and carotid artery (ΔThc) was considered to be the time between the end of the second heart sound and the dicrotic notch of the carotid waveform. The path length from the suprasternal notch to the carotid artery (Lc) was obtained using the following equation [17]:

Lc = 0.2437 × height − 18.999.

hcPWV was calculated as:

hcPWV = Lc/ΔThc.

cAIx, a measure of arterial stiffness and arterial wave reflection, was computed by dividing the amplitude of the pressure wave above the systolic shoulder by the total pulse pressure (PP) in the carotid artery [18].

We adjusted the AIx for a heart rate of 75 beats/min (AIx@75).

An applanation tonometer (Form PWV/ABI, Omron-Colin) in a neck collar adjacent to the left common carotid artery was used to measure cSBP. We calibrated the tonometer by setting the mean arterial and DBP values to be equivalent to those of the brachial artery [19].

### 2.6. Brachial BP and Heart Rate

Heart rate and normal resting BP were measured in triplicate using an automated oscillometric device (Form PWV/ABI, Fukuda-Colin, Tokyo, Japan) over the brachial artery while the participants were in the supine position. The pressure signal obtained by volume plethysmography was calibrated by equating the SBP and DBP values and then used to calculate the MBP and PP values.

### 2.7. Determination of Mean Blood Velocity (MBV) and Diameter of the Internal Carotid Artery (ICA)

While the study participants were the supine position in a quiet room maintained at a temperature of 23–25 °C, we determined MBV and the diameter of the ICA. The same study author, who was blinded to the group assignment of the participants, obtained all images. The ICA diameter and MBV were visualized by using B-mode ultrasonography with 13 MHz linear array transducers (Vivid i; GE Medical Systems, Tokyo, Japan) on the right side of the neck 1.0–1.5 cm cranial to the carotid bifurcation. The Doppler method with an insonation angle of <60° was used for MBV determinations. ICA diameters were measured using high-resolution B-mode ultrasound images, which were stored on a personal computer. Computerized image-analysis software (Scion Image Beta 4.02, National Insitutes of Health, Bethesda, MD, USA) was used to measure the ICA diameters.

### 2.8. TMT

Processing speed and flexibility in task switching and cognition were assessed using the TMT parts A (TMT-A) and B (TMT-B) [20]. In TMT-A and TMT-B, participants are asked to connect consecutive numbers (e.g., 1–2–3) and alternating numbers and letters (e.g., 1–A–2–B) in ascending order as quickly and accurately as possible to measure processing speed and “task-shifting”, respectively.

### 2.9. Serum Lipid Profile

Blood samples were collected as previously reported [21] and then mixed with 1 mL of distilled water before being centrifuged at 4000 relative centrifugal force for 15 min. Serum concentrations of fasting glucose, total cholesterol, HDL-C, HDL-C, and TG were determined using standard enzymatic techniques with intra- and inter-assay coefficients of variance of <5%.

### 2.10. IHG Training

IHG training involved the use of a handgrip dynamometer (Ohashi-Chiso, Tokyo, Japan) for 5 days per week over the 8-week study period. The handgrip dynamometer was also used to calculate the maximal voluntary contraction (MVC) before each training session. IHG training consisted of four 2-min bilateral contractions at 30% of MVC with 1-min rest periods. After the participants were familiar with the IHG training procedure, they were able to perform it at home. To confirm compliance status, the participants were given training log books for recording the dates of the exercise. Compliance status was confirmed at the end of each week, after which the participants were encouraged over the telephone to continue IHG training. All participants completed the IHG training 5 days/week for the entire 8-week study period, during which the CON group was instructed to maintain their normal lifestyle.

### 2.11. Statistical Analysis

All data are expressed as the mean ± standard error. All statistical analyses were conducted using SPSS (ver. 24; SPSS, Chicago, IL, USA). The Shapiro–Wilk test was used to verify the assumption of a normal distribution for all data, which were analyzed using two-way (group × period) repeated-measures analysis of variance. The Bonferroni–Dunn post hoc test and Pearson’s correlation were used to identify significant differences among the mean values when the F values were significant and to determine the relationships among changes in hcPWV, carotid AIx@75 (cAIx@75), and cSBP and changes in TMT-A and TMT-B, respectively. Statistical significance was set at *p* < 0.05. In addition, the interpretation of *p*-values 0.05 > *p* ≤ 0.1 was done according to the guidelines provided by Curran-Everett and Benos [22].

## 3. Results

**Figure 2:**
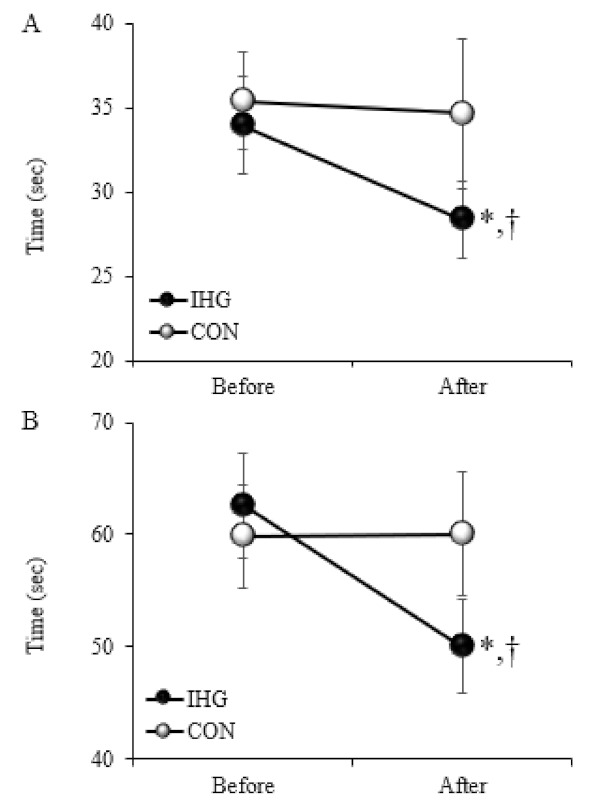
Changes in TMT-A (**A**) and TMT-B (**B**) before and after the exercise intervention in the IHG and CON groups. Values are mean ± SE; TMT, trail-making test; IHG, isometric handgrip; CON, control; *, significant difference (*p* < 0.05) from before; †, significant difference (*p* < 0.05) from the CON group.

Figure 2 shows changes in TMT-A (upper) and TMT-B (bottom) before and after the exercise intervention in the IHG and CON groups. No significant differences in TMT-A or TMT-B were observed between the two groups at baseline, whereas significant decreases in TMT-A and TMT-B were seen at 8 weeks (both *p* = 0.003) after the IHG training compared with baseline. Significant differences in TMT-A and TMT-B were found between the two groups after the exercise intervention (both *p* = 0.01).

Table 2 shows changes in hcPWV, cAIx@75, cSBP, bSBP, bracial DBP (bDBP), bMBP, brachial PP (bPP), and heart rate before and after the exercise intervention in the IHG and CON groups. No significant differences in hcPWV, cAIx@75, cSBP, bSBP, bDBP, bMBP, bPP, or heart rate were observed between the two groups at baseline, whereas significant decreases in hcPWV, cAIx@75, cSBP, and bSBP were seen at 8 weeks (*p* = 0.003, *p* = 0.003, *p* = 0.003, and *p* = 0.005, respectively) after the IHG training compared with baseline. Significant differences in hcPWV, cAIx@75, cSBP, and bSBP were found between the two groups after the exercise intervention (*p* = 0.01, *p* = 0.01, *p* = 0.01, and *p* = 0.004, respectively). No significant changes in bDBP or bMBP were seen after the exercise intervention in either group. bPP decreased significantly at 8 weeks (*p* = 0.001) after the IHG training compared with baseline. No significant difference in bPP was found between the two groups after the exercise intervention although a trend for PP to be lower in the IHG than in the CON group was observed after the exercise intervention (*p* = 0.07). In addition, no significant change in heart rate was seen after the exercise intervention in either group.

Table 3 shows the correlations between the amount of change in hcPWV (ΔhcPWV) and cAIx@75 (ΔAIx@75) and the amount of change in TMT-A (ΔTMT-A) and TMT-B (ΔTMT-B). Significant positive correlations were observed between ΔhcPWV and ΔAIx@75 and ΔTMT-A in the IHG group. On the other hand, no significant correlations were observed between ΔhcPWV and ΔAIx@75 and ΔTMT-B although a trend was seen for ΔhcPWV and ΔAIx@75 to be associated with ΔTMT-B (*p* = 0.06 and *p* = 0.05, respectively). By contrast, no significant correlations were observed between ΔhcPWV and ΔAIx@75 and ΔTMT-A or ΔTMT-B in the CON group.

Table 4 shows changes in blood flow velocity, ICA diameter, and blood flow before and after the exercise intervention in the IHG and CON groups. No significant changes in blood flow velocity, ICA diameter, or blood flow were found before or after the exercise intervention in either group.

## 4. Discussion

The key findings of this study were that hcPWV, cAIx@75, cSBP, and TMT-A and TMT-B times decreased significantly after IHG training. In addition, a significant positive correlation was identified between ΔhcPWV, cAIx@75, and cSBP and ΔTMT-A, and a tendency for a positive correlation was found between ΔhcPWV, ΔcAIx@75, and cSBP and ΔTMT-B after IHG training. To our knowledge, these results provide the first evidence that improvements in arterial function after IHG training are associated with improvements in cognitive function in older adults.

Recent meta-analyses have reported that bSBP and bDBP decrease following IHG training in all age groups (i.e., young, middle-aged, and older adults) with or without hypertension [9,11,12,23]. In addition, IHG training decreases central arterial stiffness, BP, and pulse wave reflection in middle-aged and older adults with normotension or hypertension, similar to brachial BP [10,13,24]. Consistent with previous studies, our findings demonstrated that IHG training reduces central arterial stiffness, central and peripheral BP, and pulse wave reflections. Indeed, IHG training seems to exert a beneficial effect by improving arterial function.

Two meta-analyses of data from randomized controlled trials have reported positive effects of physical exercise on cognitive function in older adults without mild cognitive impairment or dementia [25,26]. To the best of our knowledge, less is known about the effects of IHG training on cognitive function in older adults. Several studies have reported that exercise training improves cognitive function as assessed by TMT. For example, Garcia-Garro et al. [27] reported that TMT-A and TMT-B decreased by 23% and 14%, respectively, after Pilates training. Okamoto et al. [28] also reported that TMT-A and TMT-B decreased by 19% and 11%, respectively, after 20 weeks of interval walking training. In the present study, we found that TMT-A and TMT-B decreased by 16% and 20%, respectively, in older adults after IHG training. These findings provide new information on the effects of a different exercise modality, namely IHG training, on cognitive ability in older adults.

Increased central arterial stiffness is known to be associated with an increased risk of cognitive decline and impairment in older adults [7]. In fact, previous studies have reported that higher PWV predicts poor cognitive performance and a steeper cognitive decline [6,29,30,31,32]. In the present study, significant correlations were found between ΔhcPWV, ΔAIx@75, and Δcarotid BP and ΔTMT-A, and a tendency for a positive correlation was observed between ΔcfPWV, ΔAIx@75, and Δcarotid BP and ΔTMT-B in the IHG group. Therefore, IHG training appears to improve central arterial stiffness and/or pulse wave reflections, which are associated with improved cognitive ability. The established buffering capacity that accompanies reduced arterial stiffness and/or pulse wave reflections after IHG training may improve cognitive ability. Therefore, even in the absence of other types of physical activities, IHG training may improve arterial function and cognitive ability. Our results showing that IHG training leads to changes in central arterial stiffness, BP, and pulse wave reflection, which are correlated with changes in TMT-A and TMT-B, is consistent with the idea that IHG training is an important non-pharmacological therapy that plays a key clinical role in improving cardiovascular and neurological health.

Chronic cerebral hypoperfusion, which causes an inadequate supply of blood to the brain, can lead to vascular dementia [5]. Aerobic exercise increases cerebral blood flow, which is associated with improvements in cognitive function [33,34]. Therefore, increasing cerebral blood flow with exercise training, such as aerobic exercise, is important for improving cognitive function. However, our findings indicated that blood flow and ICA diameter did not change after IHG training. These results suggest that IHG training does not increase blood flow to the brain. Nonetheless, the present findings suggest that IHG training improves cognitive function in older adults; reduces microvascular damage in the brain caused by decreases in arterial stiffness, BP, and wave reflections; and exerts a beneficial effect by improving cognitive function. In fact, because blood from the left ventricle enters the brain via central elastic arteries, an elevation in arterial stiffness may induce microvascular injury in the brain [35], which can accelerate clinically evident cognitive dysfunction [36]. Long-term exposure to higher BP may injure these small vessels, leading to cognitive decline [6,31]. Hence, cognitive decline may be related to increased central arterial stiffness rather than blood flow. Therefore, these results suggest that although IHG training does not affect blood flow, it improves cognitive function by reducing arterial stiffness and pulse wave reflection. Future investigations are needed to elucidate the putative mechanisms that underlie the association between arterial stiffness and cognitive function.

IHG training is recommended for lowering BP in the guidelines of the American Heart Association (Class IIB, level of evidence C) [8]. Our results suggest that IHG training reduces central arterial stiffness and BP and improves cognitive ability, thereby extending its previously stated beneficial effects to include benefits for cognitive ability in older adults. Therefore, clinically, IHG training may help reduce the risk of vascular dementia and increase independence and quality of life in older adults. In addition, IHG training can be performed at home and has important clinical implications, such as improving cardiovascular and neurological health in older adults without drugs or the need to exercise outside. Further studies are warranted to determine the effects of IHG training on arterial and cognitive function.

This study had some limitations. First, although the magnitude of the effect on arterial stiffness, wave reflections, and cognitive function were adequate and similar to previous studies that have examined the effects of IHG on central BP in middle-aged and older adults, the sample size was small (IHG group: *n* = 11, CON group *n* = 11) [13]. Second, the participants in this study consisted of healthy older adults, which precludes generalizing our findings to patients with cardiovascular diseases and/or dementia. Third, cognitive function was assessed using only TMT-A and TMT-B. Fourth, the physical activity levels of participants were evaluated using only the International Physical Activity Questionnaire—Short Form. Finally, the exercise intervention period for this study was only 8 weeks. Further long-term, large-cohort, randomized, interventional studies are needed to establish the role of IHG training in supporting cardiovascular and brain health in older adults.

## 5. Conclusions

The present findings suggest that IHG training reduces central arterial stiffness, BP, and wave reflection, and improves cognitive function in older adults. In addition, the amount of change in cardiovascular indices (e.g., central arterial stiffness, BP, and wave reflection) was found to be correlated with the amount of change in cognitive function after IHG training. Therefore, our findings suggest that decreased arterial stiffness and wave reflections after IHG training are associated with improvements in cognitive function in older adults. IHG training may therefore be considered a non-pharmacological therapy that can improve arterial and cognitive function and thus an important strategy for preventing or improving cardiovascular diseases and/or vascular dementia in older adults.

## Figures and Tables

**Table 1:** Participants’ characteristics and serum lipids profile at baseline.

	IHG	CON
Sex (female/male)	7/4	6/5
Age (years)	76 ± 2	74 ± 2
Height (cm)	156.9 ± 2.7	159.1 ± 2.8
Weight (kg)	53.1 ± 3.3	55.4 ± 2.5
BMI (kg/m^2^)	21.4 ± 0.9	21.8 ± 0.6
Body fat (%)	24.4 ± 2.2	23.3 ± 2.0
TG (mg/dL)	94 ± 18	83 ± 9
TC (mg/dL)	215 ± 9	216 ± 9
HDL-C (mg/dL)	67 ± 4	63 ± 4
LDL-C (mg/dL)	124 ± 7	130 ± 8

Values are mean ± SE; IHG, isometric handgrip; CON, control; BMI, body mass index; TG, triglycerides; TC, total cholesterol; HDL-C, high-density lipoprotein cholesterol; LDL-C, low-density lipoprotein cholesterol.

**Table 2:** Changes in hcPWV, cAIx@75, BP, and heart rate.

	IHG		CON	
	Before	After	Before	After
hcPWV (cm/s)	1245 ± 59	1015 ± 54 **,#	1190 ± 70	1201 ± 73
cAIx@75 (%)	41 ± 3	31 ± 3 **,#	44 ± 4	47 ± 4
Carotid SBP (mmHg)	147 ± 6	136 ± 5 **,#	146 ± 4	150 ± 3
Brachial SBP (mmHg)	139 ± 5	130 ± 4 **,##	139 ± 2	140 ± 3
Brachial DBP (mmHg)	79 ± 3	77 ± 2	79 ± 2	78 ± 2
Brachial MBP (mmHg)	99 ± 3	95 ± 2	99 ± 2	99 ± 2
Brachial PP (mmHg)	61 ± 4	53 ± 3 *	59 ± 2	62 ± 2
Heart rate (beats/min)	60 ± 2	61 ± 2	59 ± 2	58 ± 2

Values are mean ± SE; IHG, isometric handgrip; CON, control; hcPWV, heart-carotid pulse wave velocity; cAIx@75, carotid augmentation index adjusted for heart rate of 75 beats/min; SBP, systolic blood pressure; DBP, diastolic blood pressure; MBP, mean blood pressure; PP, pulse pressure; *, significant (*p* < 0.05); **, significant (*p* < 0.01) difference from before; ##, significant (*p* < 0.01) difference from the control; #, significant (*p* < 0.05) difference from the control.

**Table 3:** Correlation coefficients between ΔTMT-A and ΔTMT-B as well as between ΔhcPWV and ΔcAIx@75.

	IHG		CON	
	ΔTMT-A (s)	ΔTMT-B (s)	ΔTMT-A (s)	ΔTMT-B (s)
ΔhcPWV (cm/s)	0.610 *	0.575 ‡	−0.329	0.005
ΔcAIx@75 (%)	0.604 *	0.591 †	−0.194	−0.215

Values are mean ± SE; IHG, isometric handgrip; CON, control; TMT, trail-making test; hcPWV, heart-carotid pulse wave velocity; cAIx@75, carotid augmentation index adjusted for heart rate of 75 beats/min. * *p* < 0.05, † *p* = 0.05, ‡ *p* = 0.06.

**Table 4:** Changes in blood flow velocity, ICA diameter, and blood flow.

	IHG		CON	
	Before	After	Before	After
Blood flow velocity (cm/s)	31.9 ± 3.7	35.9 ± 4.5	30.4 ± 3.4	29.2 ± 3.1
ICA diameter (mm)	5.1 ± 0.2	5.2 ± 0.3	5.0 ± 0.2	5.0 ± 0.2
Blood flow (mL/min)	414 ± 63	455 ± 76	403 ± 57	389 ± 61

Values are mean ± SE; IHG, isometric handgrip; CON, control; ICA, internal carotid artery.

## Data Availability

The data that support the findings of current study are available from the corresponding author on reasonable request.
